# Non-Invasive Assessment of SMAD4 Expression Status in Pancreatic Ductal Adenocarcinoma: Diagnostic Value of T1ρ-Weighted Imaging Combined with Clinical Parameters

**DOI:** 10.3390/diagnostics16203270

**Published:** 2026-10-09

**Authors:** Ke Li, Rui Guo, Jing Li, Qian Wu, Xiaoling Liu, Jiafei Chen, Xiaojun Wang, Wei Chen

**Affiliations:** 1 Department of Radiology, Southwest Hospital, Army Medical University (Third Military Medical University), Chongqing 400038, China; like0495@tmmu.edu.cn (K.L.); 77885410@tmmu.edu.cn (J.L.); 763671449@qq.com (X.L.); chenjiafei@tmmu.edu.cn (J.C.); 2 Department of Medical Imaging, Sichuan Corps Hospital of Chinese People’s Armed Police Force, Leshan 614000, China; 3 Institute of Hepatobiliary Surgery, Southwest Hospital, Army Medical University (Third Military Medical University), Chongqing 400038, China; ruixx032@163.com

**Keywords:** pancreatic ductal adenocarcinoma, SMAD4 protein, T1ρ-weighted imaging, magnetic resonance imaging

## Abstract

**Background/Objectives**: Pancreatic ductal adenocarcinoma (PDAC) is a malignancy with poor prognosis, where SMAD4 protein loss is closely associated with increased tumor aggressiveness and metastatic potential. Current assessment of SMAD4 expression relies on IHC of tumor tissue obtained by biopsy or resection, in which limited sampling may not represent intratumoral heterogeneity. T1ρ-weighted imaging, an emerging functional MRI technique, shows promise in characterizing tumor microstructural properties. This study aimed to evaluate the value of T1ρ imaging combined with conventional MRI sequences and clinical parameters for non-invasive prediction of SMAD4 protein expression status in PDAC. **Methods**: This retrospective single-center study included 57 patients with histologically confirmed PDAC who underwent pretreatment MRI with T1ρ imaging between September 2024 and May 2025. Immunohistochemical analysis revealed SMAD4 intact in 28 tumors (49.1%) and SMAD4 loss in 29 tumors (50.9%). T1ρ values were measured and compared between SMAD4 intact and SMAD4 loss tumors. A multiparametric predictive model incorporating T1ρ values, lymph node metastasis status, and carbohydrate antigen 19-9 (CA19-9) levels was developed. **Results**: SMAD4 intact tumors demonstrated significantly higher T1ρ values compared to SMAD4 loss tumors (96.34 ± 14.12 vs. 71.93 ± 10.24 ms, *p* < 0.001). T1ρ achieved an AUC of 0.901 (95% CI: 0.817–0.986), with 82.14% sensitivity and 89.66% specificity, for predicting SMAD4 expression status. A multiparametric model combining T1ρ values, lymph node metastasis status, and CA19-9 levels yielded a numerically higher AUC of 0.932 (95% CI: 0.862–1.000), although the difference from T1ρ alone was not statistically significant (DeLong test, *p* > 0.05). **Conclusions**: T1ρ imaging showed potential for predicting SMAD4 status in PDAC.

## 1. Introduction

Pancreatic ductal adenocarcinoma (PDAC) represents one of the most lethal malignancies worldwide. According to 2022 global cancer statistics, PDAC ranks 12th in cancer incidence but 7th in cancer-related mortality, with approximately 510,000 new cases and 467,000 deaths annually [1]. In China, PDAC incidence and mortality rates have similarly increased, with approximately 131,000 new cases and 124,000 deaths in 2022, accounting for 25.7% of global incidence [2,3]. PDAC presents enormous clinical challenges: over 80% of patients are diagnosed at advanced stages, resulting in a dismal 5-year survival rate of only 10–12% [4,5]. Despite continuous improvements in treatment modalities, patient prognosis remains extremely poor, highlighting the urgent need for novel biomarkers to guide individualized therapy [6].

SMAD4 protein serves as a key transcription factor in the TGF-β signaling pathway and plays a crucial role in PDAC pathogenesis and progression. The SMAD4 gene, located on chromosome 18q21, is one of the most frequently altered tumor suppressor genes in PDAC [7]. Studies have demonstrated that SMAD4 loss or mutation is closely associated with aggressive biological behavior in PDAC, increased metastatic potential, and development of chemotherapy resistance [8,9,10]. SMAD4 expression status serves as an important prognostic predictor in PDAC, and patients with SMAD4 loss tumors typically exhibit shorter overall survival and disease-free survival [11,12]. Therefore, accurate assessment of SMAD4 expression status is crucial for developing individualized treatment strategies. However, current SMAD4 expression detection primarily relies on immunohistochemistry (IHC) of tumor tissue specimens obtained by surgical resection or biopsy, with the same interpretation criteria applied to both, and local sampling, particularly in biopsy specimens, often fails to comprehensively reflect the overall molecular characteristics of tumors due to marked intratumoral genomic and phenotypic heterogeneity [13].

Magnetic resonance imaging (MRI) plays a pivotal role in PDAC management, but conventional MRI sequences still have limitations in predicting tumor molecular characteristics [14,15]. T1rho-weighted imaging (T1ρ imaging), as an emerging functional MRI technique, measures longitudinal relaxation time of tissues in the rotating coordinate system, enabling sensitive detection of tissue microenvironmental changes, including protein content, pH values, and water molecule motion states [16,17]. T1ρ imaging has demonstrated excellent diagnostic value and prognostic prediction capability in various tumors including gliomas, prostate cancer, and breast cancer [18,19,20,21]. Based on the advantages of T1ρ imaging and the important role of SMAD4 in PDAC, this study aims to explore the feasibility of combining T1ρ imaging, conventional MRI sequences, and clinical information for predicting SMAD4 protein expression status in PDAC, providing novel imaging evidence for imaging-based prediction of SMAD4 status of PDAC.

## 2. Materials and Methods

### 2.1. Study Participants

This retrospective single-center study was conducted at Southwest Hospital from September 2024 to May 2025. The study protocol was approved by the Institutional Review Board of Southwest Hospital and performed in accordance with the Declaration of Helsinki. Clinical and MRI data were acquired during this period as part of routine diagnostic care; formal ethical approval for retrospective analysis of these data was subsequently obtained prior to data extraction. Given the retrospective design, the requirement for written informed consent was waived by the Institutional Review Board, and all patient data were anonymized prior to analysis. A total of 72 patients diagnosed with PDAC at Southwest Hospital, Chongqing, China were initially screened for eligibility. After applying inclusion and exclusion criteria, 57 patients were ultimately enrolled in this study. Inclusion criteria were: (1) age 18–80 years; (2) histologically confirmed PDAC through surgical resection or core needle biopsy; (3) pretreatment MRI examination including T1ρ imaging and conventional sequences performed within 2 weeks prior to surgical resection or core needle biopsy; and (4) availability of adequate tumor tissue specimens for SMAD4 immunohistochemical analysis. Exclusion criteria included: (1) other pancreatic neoplasms; (2) contraindications to MRI or gadolinium-based contrast agents; (3) image quality inadequate for evaluation; (4) incomplete clinical or pathological data; (5) prior anticancer treatment including chemotherapy, radiotherapy, or targeted therapy; and (6) concomitant major systemic diseases that could interfere with study procedures or outcomes. All procedures were performed in accordance with institutional ethical standards, with appropriate safeguards to protect patients’ rights, safety, and privacy.

### 2.2. MRI Protocols

Since 2018, T1ρ imaging has been part of the routine clinical MRI protocol for pancreatic masses at Southwest Hospital. All MRI examinations were performed on a 3.0T MRI scanner (uMR 770, United Imaging Healthcare, Shanghai, China) using a multichannel phased-array body coil. Patients were positioned supine with the imaging field centered on the upper abdomen. All examinations were performed following standardized upper abdominal MRI protocols as recommended by international imaging guidelines [22]. The imaging protocol included T1-weighted imaging (T1WI), T2-weighted imaging (T2WI), diffusion-weighted imaging (DWI), T1ρ imaging, and contrast-enhanced T1WI imaging (CE-T1WI). T2WI was acquired using a respiratory-triggered fast spin-echo sequence in the coronal plane with the following parameters: repetition time/echo time (TR/TE) 1000/103.2 ms, field of view (FOV) 380 × 380 mm, matrix 320 × 256, slice thickness 4 mm with 20% gap, and voxel size 1.48 × 1.19 × 4.00 mm^3^. Axial T2WI with fat suppression was performed using TR/TE 3000/87.6 ms, FOV 400 × 300 mm, matrix 256 × 230, slice thickness 4 mm with 10% gap, and voxel size 1.74 × 1.56 × 4.00 mm^3^. T1WI was acquired in the axial plane using a dual-echo gradient-echo sequence with TR/TE 159.2/1.87 ms, FOV 400 × 320 mm, matrix 256 × 218, slice thickness 4 mm with 20% gap, flip angle 70°, and voxel size 1.84 × 1.56 × 4.00 mm^3^. DWI was performed using a single-shot echo-planar imaging sequence with TR/TE 5200/77.4 ms, FOV 380 × 300 mm, matrix 128 × 128, slice thickness 5 mm, voxel size 2.97 × 2.97 × 5.00 mm^3^, b-values of 0 and 800 s/mm^2^, and parallel imaging factor 2. T1ρ-weighted imaging was acquired using a three-dimensional gradient-echo sequence with spin-lock preparation, with parameters set according to previously validated acquisition protocols [23]. The imaging parameters were: TR/TE 7.5/3.5 ms, FOV 380 × 285 mm, matrix 208 × 146, slice thickness 4 mm, flip angle 15°, voxel size 2.61 × 1.83 × 4.00 mm^3^, spin-lock frequency 300 Hz, time of spin-lock (TSL) 0, 20, 35, and 55 ms, and parallel imaging factor 3. All four TSL-weighted images were acquired within a single breath-hold to ensure spatial co-registration and minimize respiratory motion-induced misalignment. To ensure breath-hold quality, patients underwent pre-scan breath-holding training, and images were reviewed immediately after acquisition to confirm adequate compliance. Contrast-enhanced T1WI was performed using a three-dimensional gradient-echo Dixon sequence (FACT3D) with TR/TE 10.76/1.69 ms, FOV 400 × 300 mm, matrix 208 × 146, slice thickness 4 mm, flip angle 3°, and voxel size 2.75 × 1.92 × 4.00 mm^3^. Gadolinium-based contrast agent (gadoteridol injection, Bracco, 0.1 mmol/kg) was administered intravenously at a rate of 2.0 mL/s using an automatic injector, followed by a 20 mL saline flush. All sequences were acquired during breath-holding to minimize motion artifacts.

### 2.3. Imaging Analysis

All quantitative T1ρ map analyses were performed independently by two experienced radiologists with 11 and 5 years of experience in abdominal MRI interpretation, respectively, who were not aware of clinical information and histopathological results. Images were transferred to a post-processing workstation (uOmnispace.MR, United Imaging Healthcare Co., Ltd., Shanghai, China) for quantitative analysis. For T1ρ-weighted imaging analysis, regions of interest (ROI) were manually drawn as large as possible on the axial slice showing the maximum tumor diameter, carefully avoiding vessels, necrotic areas, pancreatic ducts, and adjacent normal pancreatic tissue. T1ρ values were calculated using a monoexponential decay model: S(TSL) = S0 × exp(−TSL/T1ρ), where S(TSL) represents signal intensity at different spin-lock times, S0 is the signal intensity at TSL = 0 ms, and TSL refers to the time of spin-lock. Apparent diffusion coefficient (ADC) values were measured using the same ROI placement strategy on DWI images with b-values of 0 and 800 s/mm^2^. Additional morphological parameters including maximum tumor diameter, tumor location (head, body, or tail), and regional lymph node metastasis status were also evaluated on conventional MRI sequences. For continuous variables (T1ρ values, ADC values, and tumor diameter), the mean values of measurements from both observers were used for statistical analysis. For categorical variables (tumor location and lymph node status), discrepancies between the two observers were resolved through consensus discussion. The original measurements from both observers were preserved for subsequent inter-observer agreement analysis.

### 2.4. Clinical, Laboratory, and Immunohistochemistry Data Analysis

Clinical and laboratory data were collected for all enrolled patients, including demographic information such as sex, age, and body mass index (BMI). Laboratory parameters included carcinoembryonic antigen (CEA), carbohydrate antigen 19-9 (CA19-9), carbohydrate antigen 125 (CA125), gamma-glutamyl transpeptidase (GGT), alanine aminotransferase (ALT), and total bilirubin (TBIL). All clinical variables and MRI examinations were obtained within 2 weeks prior to surgical resection or core needle biopsy to ensure temporal consistency.

SMAD4 status was determined on surgical specimens in patients who underwent resection, and on multi-site core needle biopsy specimens in the 5 patients who did not undergo surgery. Immunohistochemical staining for SMAD4 protein expression was performed on all tumor specimens using the automated immunohistochemical detection system (Leica Bond Max System, Leica Biosystems, Wetzlar, Germany) with monoclonal antibodies against SMAD4 (clone B8, Santa Cruz Biotechnology, Dallas, TX, USA). SMAD4 expression was assessed using a binary classification system: SMAD4 loss was defined as complete absence of nuclear staining in tumor cells while adjacent non-tumor cells (stromal cells, blood vessels, or normal pancreatic tissue) retained positive nuclear staining as internal positive controls; SMAD4 intact was defined as tumor cells showing nuclear staining comparable to internal control tissues. Notably, any discernible nuclear positivity in tumor cells, even if weaker than the internal control, was classified as SMAD4 intact [24,25]. Two experienced pathologists with 6 and 14 years of pancreatic pathology experience independently evaluated all slides without knowledge of clinical data or imaging findings. Discordant cases were resolved through consensus review. Known positive control tissues and phosphate-buffered saline negative controls were included in each staining batch for quality assurance. Tumors were grouped by IHC-determined SMAD4 status (intact or loss).

### 2.5. Statistical Analysis

Statistical analyses were performed using R software (version 4.3.0, R Foundation for Statistical Computing, Vienna, Austria) and MedCalc statistical software (version 20.0, MedCalc Software Ltd., Ostend, Belgium). Quantitative variables are presented as mean ± standard deviation or median (interquartile range). Categorical variables are expressed as frequencies and percentages. Interobserver agreement was assessed using intraclass correlation coefficient (ICC) with a two-way random effects model. Continuous variables were compared using Student’s *t*-test or Mann–Whitney U test as appropriate. Categorical variables were compared using chi-square test or Fisher’s exact test. Receiver operating characteristic (ROC) curve analysis was performed to evaluate diagnostic performance, with calculation of area under the curve (AUC), sensitivity, specificity, positive predictive value, and negative predictive value with 95% confidence intervals. The optimal cutoff value was determined by maximizing the Youden index. AUC was compared using the DeLong test. Internal validation was performed using bootstrap resampling (1000 resamples), in which model fitting was repeated in each resample; the optimism-corrected AUC and its 95% CI (percentile method) were calculated. Restricted cubic spline analysis with three knots was conducted using logistic regression to explore non-linear relationships. Threshold effects were assessed using a two-piecewise logistic regression model, with the inflection point determined by maximum likelihood and tested against a one-line model (log-likelihood ratio test). Logistic regression was used for the combined model and subgroup analyses, with interaction effects tested using interaction terms. Due to an empty cell in the CA19-9–SMAD4 table causing quasi-complete separation, the combined model was refitted using Firth’s penalized logistic regression. A two-sided *p* value < 0.05 was considered statistically significant.

## 3. Results

### 3.1. Clinical Characteristics

**Figure 1:**
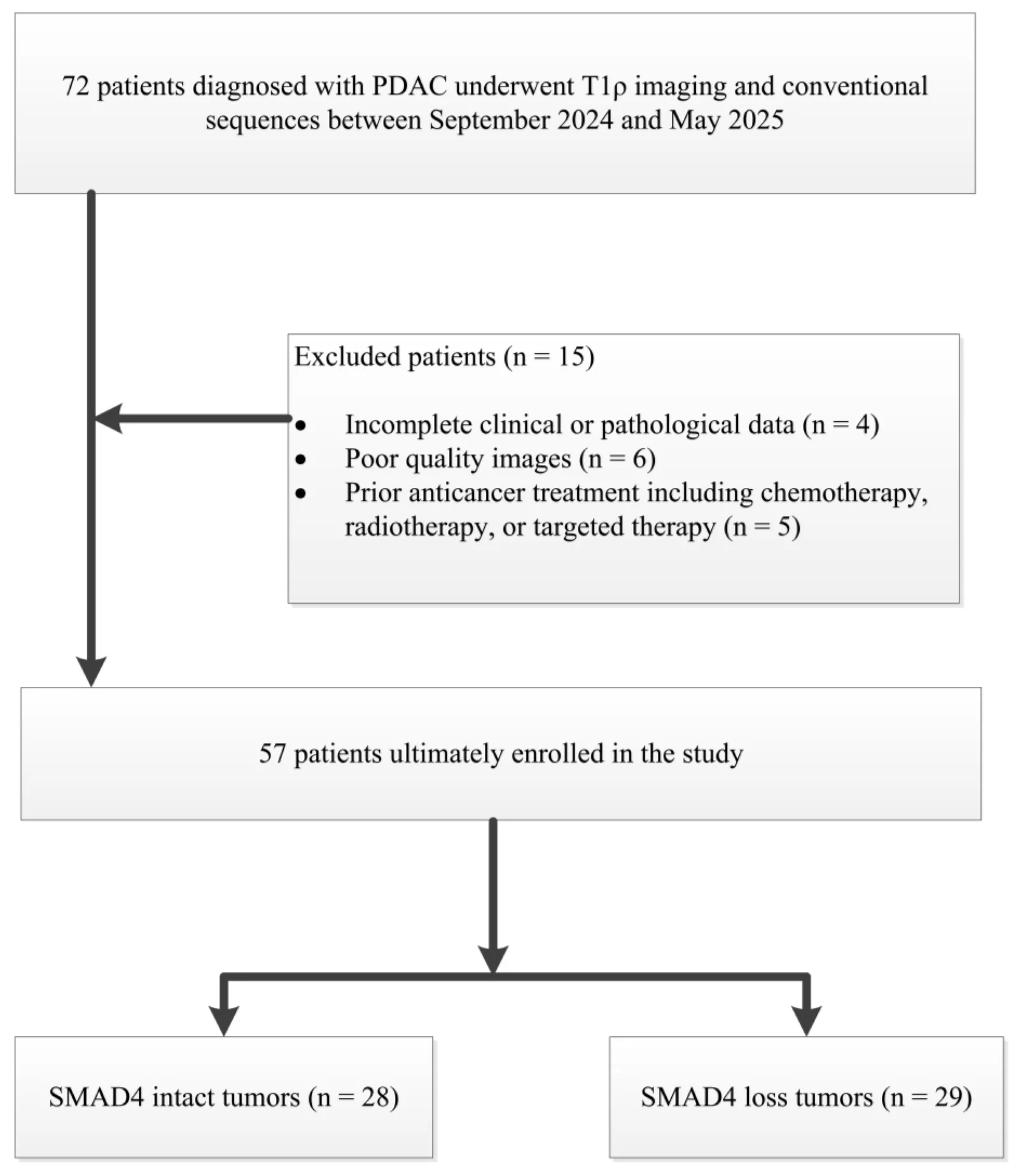
The enrolled patient flowchart.

A total of 57 patients were ultimately enrolled in this study (Figure 1). The mean age was 61.4 ± 9.7 years, 31 patients (54.4%) were male and 26 (45.6%) were female. The mean BMI was 22.8 ± 3.7 kg/m^2^. Thirty-eight tumors (66.7%) were located in the pancreatic head, and 19 (33.3%) in the pancreatic body or tail. The mean maximum tumor diameter was 3.2 ± 1.3 cm.

SMAD4 IHC revealed SMAD4 intact in 28 tumors (49.1%) and SMAD4 loss in 29 tumors (50.9%). Specimens were obtained by surgical resection in 52 patients (91.2%) and by multi-site core needle biopsy in five patients (8.8%).

### 3.2. Clinical and Imaging Characteristics According to SMAD4 Status

**Figure 2:**
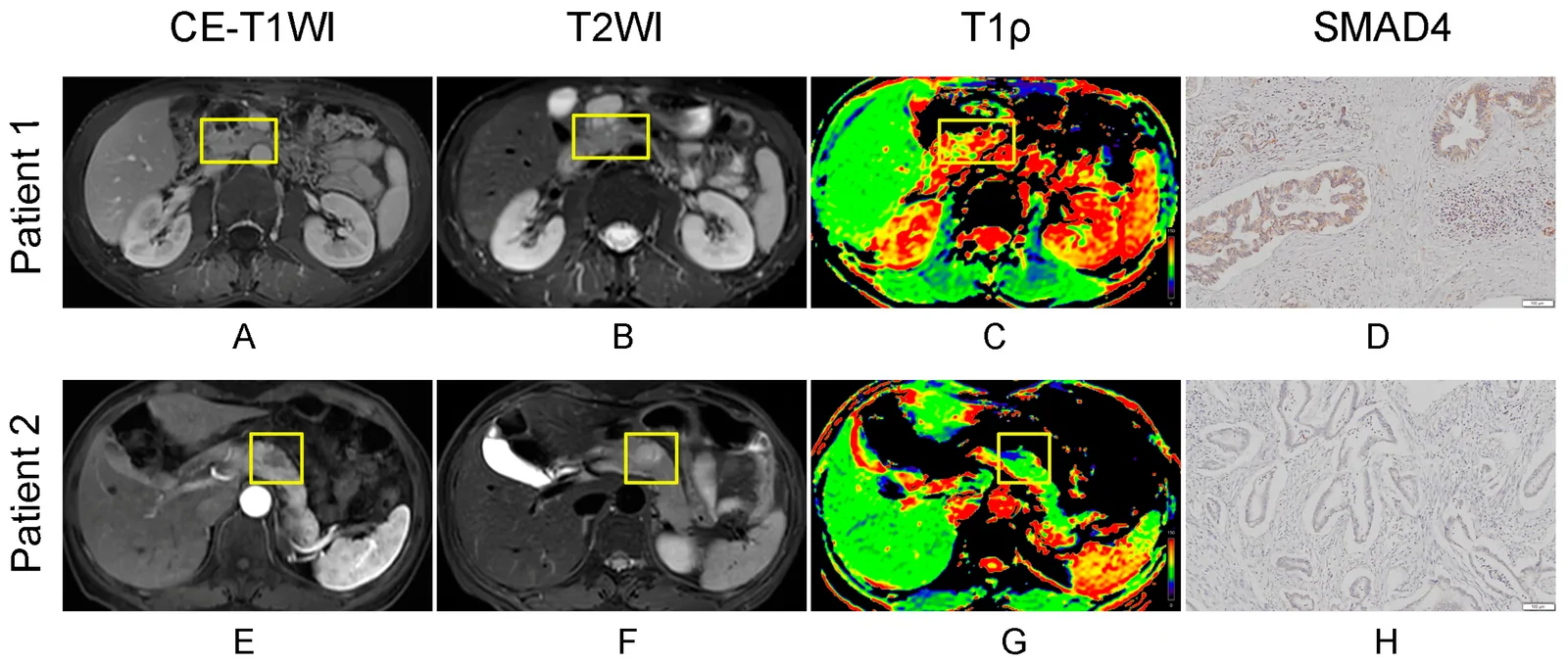
Representative MR images and SMAD4 immunohistochemical staining of PDAC in two patients. Patient 1 (**A**–**D**): A 61-year-old male with PDAC in the pancreatic head. (**A**) CE-T1WI, (**B**) T2WI, (**C**) T1ρ imaging showing a relatively high T1ρ value (110 ms), and (**D**) immunohistochemical staining showing a SMAD4 intact tumor. Patient 2 (**E**–**H**): A 63-year-old female with PDAC in the pancreatic body. (**E**) CE-T1WI, (**F**) T2WI, (**G**) T1ρ imaging showing a relatively low T1ρ value (71 ms), and (**H**) immunohistochemical staining showing a SMAD4-loss tumor. The color bar in panels C and G represents T1ρ values ranging from 0 to 150 ms. original magnification ×200; scale bar = 100 μm.

Interobserver agreement for quantitative measurements was good to excellent, with ICC values of 0.89, 0.86, and 0.94 for T1ρ, ADC, and maximum tumor diameter measurements, respectively. The detailed comparison between SMAD4 intact and SMAD4 loss tumors is presented in Table 1. Representative images are shown in Figure 2. No significant differences were observed in clinical characteristics, including age (60.64 ± 9.76 vs. 62.17 ± 9.74 years, *p* = 0.556), BMI (22.75 ± 3.09 vs. 22.85 ± 4.23 kg/m^2^, *p* = 0.918), maximum tumor diameter (3.07 ± 1.19 vs. 3.33 ± 1.31 cm, *p* = 0.444), sex distribution (*p* = 0.903), and tumor location (*p* = 0.349).

Regarding imaging characteristics, while ADC values showed no significant difference between SMAD4 intact and SMAD4 loss tumors (1.560 ± 0.216 vs. 1.532 ± 0.194 × 10^−3^ mm^2^/s, *p* = 0.607), T1ρ values were significantly higher in the SMAD4 intact than in SMAD4 loss tumors (96.34 ± 14.12 vs. 71.93 ± 10.24 ms, *p* < 0.001). Additionally, lymph node metastasis was significantly more frequent in the SMAD4 loss tumors (55.17% vs. 25.00%, *p* = 0.020).

Among laboratory parameters, CA19-9 demonstrated a significant difference, with normal values (<25 U/mL) observed in 21.43% of patients with SMAD4 intact tumors compared to 0% in patients with SMAD4 loss tumors (*p* = 0.008). Other tumor markers including CEA, CA125, and liver function parameters showed no significant differences between SMAD4 intact and SMAD4 loss tumors.

### 3.3. Diagnostic Performance for Predicting SMAD4 Status

**Figure 3:**
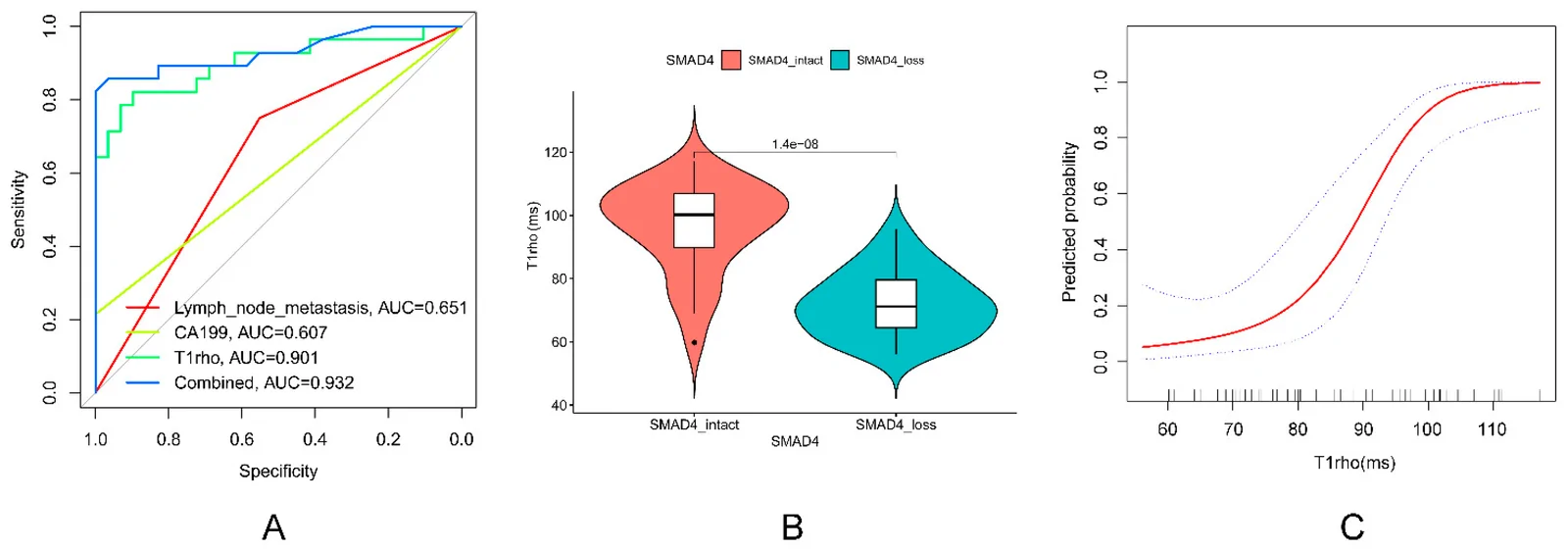
Diagnostic performance of T1ρ values for SMAD4 status prediction in PDAC. (**A**) ROC curves for lymph node metastasis, CA 19-9, T1ρ, and the combined multiparametric model. (**B**) Violin plots illustrating the distribution of T1ρ values between SMAD4 intact and SMAD4 loss tumors. (**C**) Restricted cubic spline curve showing the relationship between T1ρ values and the predicted probability of SMAD4 intact status. The red solid line represents the fitted curve, and the purple dotted lines represent the 95% confidence interval. Tick marks along the x-axis indicate the T1ρ values of individual patients.

SMAD4 intact status was defined as the positive class for all subsequent diagnostic performance analyses. The diagnostic performance of parameters for predicting SMAD4 expression status is presented in Table 2 and Figure 3A. T1ρ achieved an AUC of 0.901 (95% CI: 0.817–0.986) at the optimal cutoff of 85.87 ms with 82.14% sensitivity, 89.66% specificity, and 85.96% accuracy. Lymph node metastasis showed an AUC of 0.651 (95% CI: 0.507–0.795) with 75.00% sensitivity and 55.17% specificity. CA 19-9 demonstrated an AUC of 0.607 (95% CI: 0.459–0.755) with 21.43% sensitivity and 100.00% specificity.

The combined model incorporating T1ρ values, lymph node metastasis status, and CA19-9 levels yielded an AUC of 0.932 (95% CI: 0.862–1.000) with 85.71% sensitivity, 96.55% specificity, and 91.23% accuracy. DeLong test revealed no significant difference in AUC between T1ρ alone and the combined model (*p* > 0.05). After bootstrap internal validation, the optimism-corrected AUCs were 0.899 (95% CI, 0.802–0.972) for T1ρ and 0.919 (95% CI, 0.841–0.982) for the combined model. The refitted Firth model was:

logit(P)=−7.517+0.120×T1ρ−2.249×CA19−9−1.197×Lymphnodemetastasis

where *P* is the predicted probability of SMAD4 intact status; T1ρ (ms) is continuous; CA19-9 is coded 0 (<25 U/mL) or 1 (≥25 U/mL); and lymph node metastasis is coded 0 (absent) or 1 (present). T1ρ remained independently associated with SMAD4 intact status (OR = 1.13, 95% CI: 1.06–1.20, *p* < 0.001), whereas CA19-9 (OR = 0.11, 95% CI: 0.01–2.32, *p* = 0.113) and lymph node metastasis (OR = 0.30, 95% CI: 0.06–1.49, *p* = 0.094) were not significant.

### 3.4. T1ρ Distribution Characteristics and Threshold Effect Analysis

Figure 3B illustrates the T1ρ value distributions between SMAD4 intact and loss tumors, with SMAD4 intact tumors showing higher values and broader distribution range. Restricted cubic spline analysis (Figure 3C) revealed a non-linear relationship between T1ρ values and SMAD4 expression status. Linear analysis showed a significant association (*p* < 0.001). Non-linear analysis identified an inflection point at 95.6 ms, with a significant association below this point (*p* = 0.035) but not above (*p* = 0.789); the log-likelihood ratio test confirmed a threshold effect (*p* = 0.034). This inflection point describes the shape of the relationship and is distinct from the ROC cutoff.

### 3.5. Subgroup Analysis and Interaction Effects

**Figure 4:**
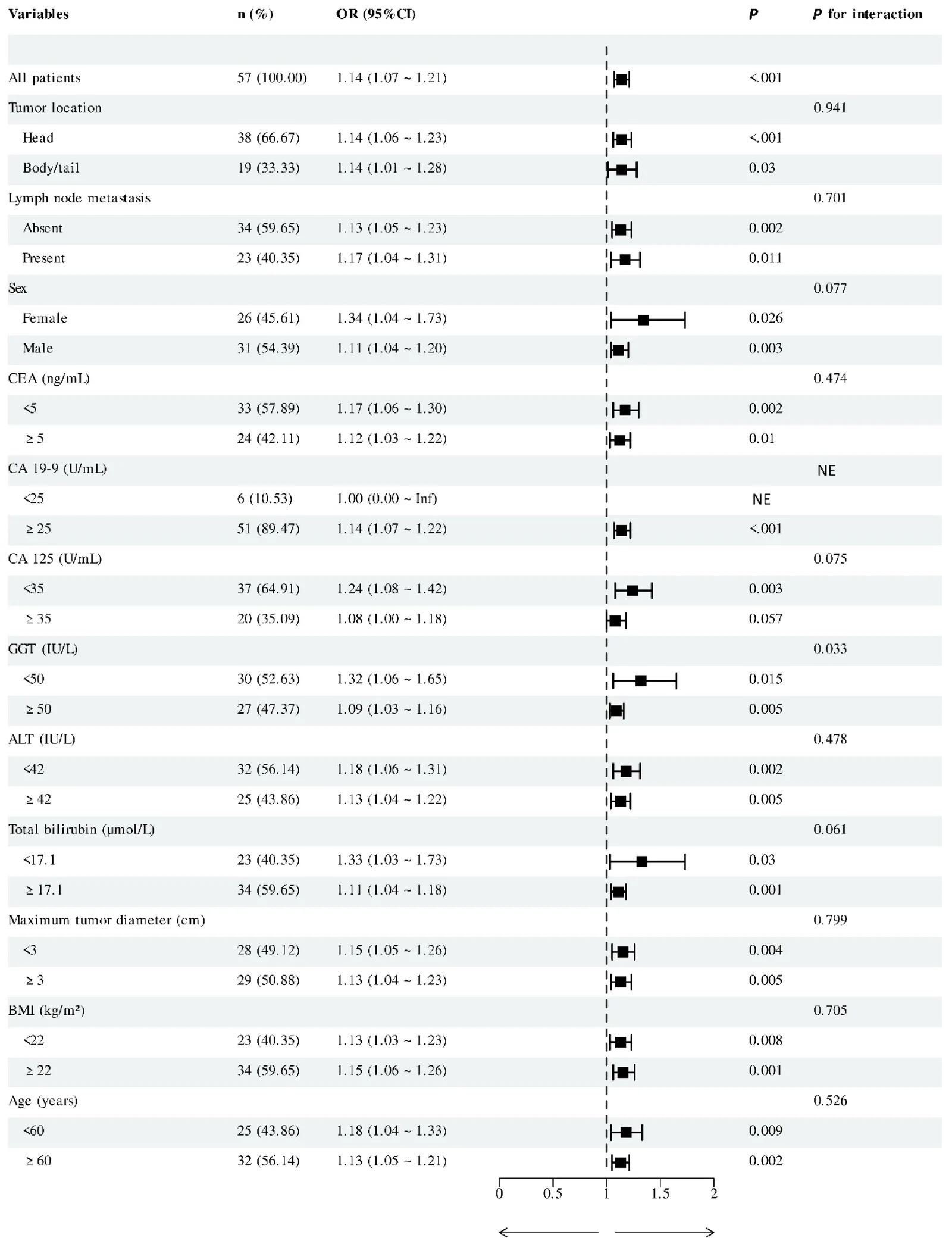
Forest plot of the association between T1ρ values and SMAD4 intact status across subgroups stratified by tumor location, lymph node metastasis, sex, age, BMI, maximum tumor diameter, and laboratory parameters (CEA, CA19-9, CA125, GGT, ALT, and total bilirubin), with *p* values for interaction. NE, not estimable.

Subgroup analysis showed generally consistent T1ρ performance across clinical characteristics (Figure 4). T1ρ was significantly associated with SMAD4 expression in all subgroups (all *p* < 0.05), except for CA125 ≥35 U/mL (*p* = 0.057) and CA19-9 < 25 U/mL, which was not estimable because all six patients had SMAD4 intact tumors. Among the 12 interaction tests, only GGT was significant (*p* = 0.033), which should be regarded as exploratory; no other interactions were significant (all *p* > 0.05).

## 4. Discussion

In this retrospective study, we demonstrated that T1ρ imaging provides valuable information for imaging-based prediction of SMAD4 expression status in PDAC. Our findings revealed significant differences in T1ρ values between SMAD4 intact and SMAD4 loss tumors. These differences were generally consistent across exploratory subgroup analyses by tumor location and size, among other clinical variables. These differences may reflect underlying differences in tumor microenvironment and molecular characteristics associated with SMAD4 status. Notably, conventional morphological features and ADC values showed no significant differences between SMAD4 intact and SMAD4 loss tumors, whereas T1ρ achieved substantial discriminative performance (AUC = 0.901), suggesting its sensitivity to SMAD4-related molecular and microstructural alterations. Furthermore, the integration of T1ρ values with lymph node metastasis status and CA19-9 levels yielded a numerically higher AUC (0.932 vs. 0.901).

T1ρ imaging measures the spin-lattice relaxation time in the rotating frame, which is particularly sensitive to slow molecular motions and chemical exchanges in the low-frequency range (0–10 kHz) [26]. This unique sensitivity allows T1ρ to detect changes in tissue macromolecular content, water binding, and pH alterations that may not be captured by conventional MRI sequences [27]. Historically, T1ρ imaging has been predominantly applied in musculoskeletal systems, where it has demonstrated value in detecting early biochemical changes in cartilage degradation, intervertebral disk degeneration, and muscle pathologies [28,29]. More recently, T1ρ applications have expanded into oncological imaging, with favorable results in glioma grading [18], breast lesion characterization [20,30], and prostate cancer detection [21], suggesting its capability to characterize tumor properties and potentially reflect underlying molecular features across multiple organ systems. This broader oncological applicability provided the rationale for extending T1ρ imaging to PDAC in the present study, in which we observed a significant difference in T1ρ values according to SMAD4 expression status (96.34 ms vs. 71.93 ms, *p* < 0.001), suggesting that T1ρ may also be informative in PDAC.

SMAD4 is the central mediator of canonical TGF-β signaling [31,32]. Its loss disrupts this pathway and redirects TGF-β signaling through non-canonical routes, including MAPK, PI3K/AKT, and Rho-like GTPases [33,34], and has been associated with increased stromal deposition, altered extracellular matrix composition, and changes in tissue hydration and pH [35,36]. SMAD4 loss has also been linked to epithelial–mesenchymal transition [7,37,38] and to a shift toward aerobic glycolysis with lactate-related acidification [7,39]. Because T1ρ is sensitive to macromolecular content, water binding, and proton exchange [26,27], these alterations could plausibly contribute to the lower T1ρ values observed in SMAD4 loss tumors.

The broader T1ρ distribution in SMAD4 intact tumors (Figure 3B) may reflect greater variability in stromal composition, whereas SMAD4 loss has been associated with reduced immune cell infiltration [9]. These interpretations remain speculative, as stromal, immune, and metabolic features were not assessed in this study, and they require histopathological correlation in future work.

Current clinical detection of SMAD4 status in PDAC relies predominantly on tissue-based methods. Immunohistochemistry on tissue specimens remains the reference method for determining SMAD4 status, given its well-established clinicopathological significance [40], but it is inherently a postoperative assessment and thus cannot inform preoperative treatment planning. Preoperative assessment using endoscopic ultrasound-guided fine-needle aspiration/biopsy (EUS-FNA/FNB) remains challenged by sampling variability and occasionally insufficient tissue yield for comprehensive molecular profiling, underscoring the need for standardized tissue acquisition protocols to support genomic testing [41]. Additionally, these invasive procedures carry inherent risks including bleeding, infection, and potential tumor seeding [42]. These limitations highlight the need for imaging-based approaches to predict SMAD4 status. Our findings could help address this limitation: T1ρ imaging, integrated into standard pretreatment MRI protocols, achieved high diagnostic performance (AUC 0.901–0.932) for predicting SMAD4 status in this cohort, without the need for invasive tissue sampling.

Several studies have evaluated imaging-based prediction of SMAD4 expression status. Attiyeh et al. (2019) demonstrated that CT-based radiomic features were associated with SMAD4 genotype and tumor stromal content in resectable PDAC [43]. Hinzpeter et al. (2022) further showed that CT radiomics correlated with driver gene mutations, including SMAD4, providing an imaging-based avenue for radiogenomic profiling in PDAC [44]. More recently, Li et al. (2024) reported that preoperative contrast-enhanced MRI features, combined with clinical parameters such as CA19-9 level, common bile duct dilation, and rim enhancement, could effectively identify SMAD4-mutated PDAC [25]. These approaches demonstrate the potential of imaging-based prediction while indicating room for improvement in diagnostic accuracy.

Our study suggests that T1ρ imaging may provide valuable information for imaging-based prediction of SMAD4 status. We observed significant differences in T1ρ values between SMAD4 intact and SMAD4 loss tumors (96.34 ± 14.12 ms vs. 71.93 ± 10.24 ms, *p* < 0.001), suggesting that T1ρ may reflect tissue differences related to SMAD4 status. The diagnostic performance of T1ρ parameters alone was high with an AUC of 0.901 (95% CI: 0.817–0.986), sensitivity of 82.14%, and specificity of 89.66%. Furthermore, our multiparametric approach combining T1ρ values with lymph node metastasis status and CA19-9 levels achieved a numerically higher AUC (0.932) than T1ρ alone (0.901), although this difference did not reach statistical significance (DeLong test, *p* > 0.05), suggesting potential benefits of a multivariable logistic regression model combining T1ρ, lymph node status and CA19-9. Our findings suggest that T1ρ imaging may have clinical relevance in PDAC management. Given that SMAD4 loss correlates with increased metastatic potential and poorer prognosis, pretreatment identification of SMAD4 status could facilitate personalized treatment planning. Patients with SMAD4 loss tumors, who face a higher risk of metastatic recurrence, might benefit from more intensive postoperative surveillance and individualized adjuvant treatment strategies within the framework of precision oncology for PDAC [45]. Additionally, as targeted therapies against pathways dysregulated by SMAD4 loss continue to emerge, imaging-based prediction of SMAD4 status could support treatment selection [7].

Despite these results, several limitations of our study warrant consideration. First, this was a single center retrospective study with a relatively small sample size, which may introduce selection bias and limit statistical power. In addition, SMAD4 status in five patients (8.8%) was determined on core needle biopsy rather than surgical specimens, and ROI measurement on a single slice may not fully capture intratumoral heterogeneity. Second, the combined model and the T1ρ cutoff were derived in the same cohort. Although bootstrap internal validation showed minimal optimism, external validation is still needed. Independent, preferably multicenter prospective cohorts are needed to confirm the model’s diagnostic accuracy and generalizability across patient populations and imaging platforms. Third, while we showed the potential of T1ρ imaging for prediction of SMAD4 status, technical challenges remain in standardizing T1ρ acquisition protocols across different MRI vendors and field strengths. The reproducibility of T1ρ measurements may be affected by factors such as B0 and B1 field inhomogeneities, particularly in anatomically complex regions like the pancreas where susceptibility artifacts can be prominent. Additionally, further investigation of the biological mechanisms underlying the association between T1ρ signal characteristics and SMAD4 expression would enhance our understanding of the observed imaging phenotype. Future work should address these limitations by incorporating advanced motion correction techniques, developing standardized T1ρ acquisition and post-processing methods, and establishing multicenter validation cohorts with comprehensive molecular profiling to strengthen the clinical applicability of T1ρ imaging for molecular stratification of PDAC.

## 5. Conclusions

In conclusion, this preliminary study suggests that T1ρ imaging has potential for predicting IHC-defined SMAD4 status in PDAC, achieving high diagnostic performance (AUC = 0.901) that was numerically, though not statistically, improved when combined with lymph node metastasis status and CA19-9 levels (AUC = 0.932). Pending validation in larger, multicenter cohorts, this imaging-based approach may facilitate molecular stratification and support personalized treatment planning for patients with PDAC.

## Figures and Tables

**Table 1:** Comparison of clinical and imaging characteristics between SMAD4 intact and SMAD4 loss tumors.

Parameters	SMAD4 Intact (*n* = 28)	SMAD4 Loss (*n* = 29)	*p*-Value
**Clinical characteristics**			
Age (years)	60.64 ± 9.76	62.17 ± 9.74	0.556
BMI (kg/m^2^)	22.75 ± 3.09	22.85 ± 4.23	0.918
Maximum tumor diameter (cm)	3.07 ± 1.19	3.33 ± 1.31	0.444
Sex			0.903
Female	13 (46.43)	13 (44.83)	
Male	15 (53.57)	16 (55.17)	
Tumor location			0.349
Head	17 (60.71)	21 (72.41)	
Body/tail	11 (39.29)	8 (27.59)	
**Imaging characteristics**			
ADC (×10^−3^ mm^2^/s)	1.560 ± 0.216	1.532 ± 0.194	0.607
T1ρ (ms)	96.34 ± 14.12	71.93 ± 10.24	<0.001 *
Lymph node metastasis			0.020 *
Absent	21 (75.00)	13 (44.83)	
Present	7 (25.00)	16 (55.17)	
**Laboratory parameters**			
CEA (ng/mL)			0.337
<5	18 (64.29)	15 (51.72)	
≥5	10 (35.71)	14 (48.28)	
CA 19-9 (U/mL)			0.008 *
<25	6 (21.43)	0 (0.00)	
≥25	22 (78.57)	29 (100.00)	
CA 125 (U/mL)			0.117
<35	21 (75.00)	16 (55.17)	
≥35	7 (25.00)	13 (44.83)	
GGT (IU/L)			0.503
<50	16 (57.14)	14 (48.28)	
≥50	12 (42.86)	15 (51.72)	
ALT (IU/L)			0.881
<42	16 (57.14)	16 (55.17)	
≥42	12 (42.86)	13 (44.83)	
Total bilirubin (μmol/L)			0.872
<17.1	11 (39.29)	12 (41.38)	
≥17.1	17 (60.71)	17 (58.62)	

Data are presented as mean ± standard deviation for continuous variables and as number (percentage), *n* (%), for categorical variables. * *p* < 0.05 was considered statistically significant.

**Table 2:** Diagnostic performance of different parameters for predicting SMAD4 expression status in PDAC.

Parameter	AUC	95% CI	Sensitivity (%)	Specificity (%)	Accuracy (%)	PPV (%)	NPV (%)
Lymph node metastasis	0.651	0.507–0.795	75.00	55.17	64.91	61.76	69.57
CA 19-9	0.607	0.459–0.755	21.43	100.00	61.40	100.00	56.86
T1ρ	0.901	0.817–0.986	82.14	89.66	85.96	88.46	83.87
Combined	0.932	0.862–1.000	85.71	96.55	91.23	96.00	87.50

CI, confidence interval; PPV, positive predictive value; NPV, negative predictive value.

## Data Availability

The data are not publicly available due to privacy restrictions but are available from the corresponding author upon reasonable request.

## Abbreviations

The following abbreviations are used in this manuscript:

PDAC	Pancreatic ductal adenocarcinoma
MRI	Magnetic resonance imaging
ADC	Apparent diffusion coefficient
ROC	Receiver operating characteristic
AUC	Area under the curve
CI	Confidence interval
CA19-9	Carbohydrate antigen 19-9
BMI	Body mass index
FOV	Field of view
ROI	Region of interest
ICC	Intraclass correlation coefficient
CEA	Carcinoembryonic antigen
CA125	Carbohydrate antigen 125
GGT	Gamma-glutamyl transpeptidase
ALT	Alanine aminotransferase
TBIL	Total bilirubin
TGF-β	Transforming growth factor-beta
